# Soyasaponin I Improved Neuroprotection and Regeneration in Memory Deficient Model Rats

**DOI:** 10.1371/journal.pone.0081556

**Published:** 2013-12-04

**Authors:** Sung-Woon Hong, Hwon Heo, Jeong-hwa Yang, Maeum Han, Dong-Hyun Kim, Yunhee Kim Kwon

**Affiliations:** 1 Department of Life and Nanopharmaceutical Sciences, Kyung Hee University, Hoegi-dong, Dongdaemoon-gu, Seoul, Republic of Korea; 2 Department of Biology, College of Sciences, Kyung Hee University, Hoegi-dong, Dongdaemoon-gu, Seoul, Republic of Korea; University of Victoria, Canada

## Abstract

Soy (*Glycine Max* Merr, family Leguminosae) has been reported to possess anti-cancer, anti-lipidemic, estrogen-like, and memory-enhancing effects. We investigated the memory-enhancing effects and the underlying mechanisms of soyasaponin I (soya-I), a major constituent of soy. Impaired learning and memory were induced by injecting ibotenic acid into the entorhinal cortex of adult rat brains. The effects of soya-I were evaluated by measuring behavioral tasks and neuronal regeneration of memory-deficient rats. Oral administration of soya-I exhibited significant memory-enhancing effects in the passive avoidance, Y-maze, and Morris water maze tests. Soya-Ι also increased BrdU incorporation into the dentate gyrus and the number of cell types (GAD67, ChAT, and VGluT1) in the hippocampal region of memory-deficient rats, whereas the number of reactive microglia (OX42) decreased. The mechanism underlying memory improvement was assessed by detecting the differentiation and proliferation of neural precursor cells (NPCs) prepared from the embryonic hippocampus (E16) of timed-pregnant Sprague-Dawley rats using immunocytochemical staining and immunoblotting analysis. Addition of soya-Ι in the cultured NPCs significantly elevated the markers for cell proliferation (Ki-67) and neuronal differentiation (NeuN, TUJ1, and MAP2). Finally, soya-I increased neurite lengthening and the number of neurites during the differentiation of NPCs. Soya-Ι may improve hippocampal learning and memory impairment by promoting proliferation and differentiation of NPCs in the hippocampus through facilitation of neuronal regeneration and minimization of neuro-inflammation.

## Introduction

Many patients with various neurological diseases, including Alzheimer’s disease (AD), Parkinson’s disease, epilepsy, depression, and cerebral ischemia, suffer from variable degrees of learning and memory impairment [1,2]. Neuronal cell death is common in degenerative neurological diseases and occurs throughout the brain regions. Some neuronal loss may be replaced by adult neurogenesis in the subventricular zone (SVZ) of the lateral ventricle and in the subgranular zone (SGZ) of the hippocampus. Neural precursor cells (NPCs) in the SVZ migrate into the olfactory area and the sites of the neuronal cell death and differentiate to replace them, while NPCs in the SGZ migrate to and regenerate the granular cell layer of the hippocampus which governs the formation of memory [1,2,5]. In case of AD patients, neuro-degeneration is indicated by serious neuronal cell death in the cerebral cortex and hippocampus. It is caused by depositions of post-translationally misprocessed proteins, such as β-amyloid and tau, that are found in senile plaques and neurofibrillary tangles. This process is accompanied by memory loss and abnormal behavior. Defects in neurogenesis, including proliferation and differentiation of NPCs may accelerate neuronal loss in the brain of AD patients [2-4]. 

Although the role of neurogenesis in other neurological diseases that cause learning and memory impairment is under investigation, adult neurogenesis is reported to form and modulate learning and memory and may facilitate recovery of memory dysfunction in humans [1,5-7]. Adult neurogenesis, the capacity to generate new neurons, occurs continuously in the adult mammalian brain throughout life [5,6]. In particular, it has been demonstrated that neurogenesis in the dentate gyrus (DG) of the hippocampus and hippocampal learning and memory function are strongly correlated. For example, both voluntary exercise and exposure to enriched environments increase neurogenesis and enhance performance in spatial learning and memory [2,7-9]. On the other hand, suppression of neurogenesis in the DG by X-irradiation impairs hippocampus-dependent learning and memory formation [10]. A reduction in cell proliferation in the SGZ of the DG and neurodegeneration is induced in pathological conditions, exposure to chronic stresses [11], and aging [6,12,13]. DG-specific knockdown rats, with transgenic inhibition of adult-born granule cells, show impairment of long-term spatial memory [14]. Additionally, the maturation process of differentiating newly born neurons plays an important role in learning and memory [14].

Soy (*Glycine Max* Merr., family Leguminosae) is widely used as an ingredient in many foods and has been reported to show anti-cancer, anti-oxidant, anti-inflammatory, anti-lipidemic, and estrogen-like effects [15,16]. Recently, it has been shown that soy also has learning- and memory-enhancing effects [16-21]. Soy contains many phytochemicals, including isoflavones and saponins [15]. Also, isoflavones have been reported to exhibit memory-enhancing, anti-inflammatory, and phytoestrogenic effects [16-21]. In contrast, soyasaponins have anti-colitic, anti-tumor, hepatoprotective, and estrogen-like effects [22-24]. Soyasapogenol B, a metabolite of soyasaponin I has been reported to inhibit proliferation of human breast cancer cells [24]. However, it is not yet clear whether soyasaponins have learning- and memory-enhancing effects.

In this study, we isolated two major saponins from soybeans and investigated their learning- and memory-enhancing effects. Among the saponins tested, soyasaponin I (soya-I) strongly ameliorated learning and memory behaviors in memory deficient model rats. We investigated how soya-I enhances learning and memory by examining neuronal regeneration in the hippocampus of the adult model rats and proliferation and differentiation of NPCs cultured from the rat hippocampus. 

## Materials and Methods

### Materials

Dulbecco’s modified eagle medium-F12 media, Ca^+2^/Mg^+2^-free Hank’s balanced salt solution), basic fibroblast growth factor (bFGF), trypsin-ethylenediaminetetraacetic acid , secondary antibodies and L-glutamine were purchased from Invitrogen (Carlsbad, CA, USA). Primary antibodies were purchased from Abcam (Cambridge, UK), Chemicon (Billerica, MA, USA), and Serotec (Kidlington, UK). Secondary antibodies were purchased from Jackson (West Grove, PA, USA). Ibotenic acid (IBO), poly-L-ornithine, progesterone, D-(+)-Glucose, putrescine, apo-transferrin, insulin, fibronectin, 4-(2-hydroxyethyl)-1-piperazineethanesulfonic acid , phenol red, radioimmuno-precipitation assay buffer, phosphate buffered saline (PBS), phosphatase inhibitor cocktail, a protease inhibitor cocktail, dimethyl sulfoxide, paraformaldehyde (PFA) and Tween 80 were purchased from Sigma (St. Louis, MO, USA). Soyasaponins, including soya-Ι (purity, >95%), were obtained as previously reported (Chang et al., 2009, [22]. All other materials were obtained from normal commercial sources and were of the highest grade available. 

### Animals

Male Sprague Dawley rats (200 - 250 g) were obtained from the Orient Animal Breeding Center (a branch of Charles River Laboratories, Gyunggi-do, Korea). Rats were randomly housed 4 or 5 per cage for at least 1 week of habituation before starting the experiments, allowed access to water and food *ad libitum*, and maintained under a constant temperature (23±1 °C), humidity (60±10 %), and a 12-hour light/dark cycle (light on 7:00-19:00 hour). After the surgical procedure, rats were group separated and housed 2 or 3 per cage to avoid social stress. Male rats were used for all the experiments, so we do not know differences with females. All experiments were performed in accordance with the NIH and the Kyung Hee University guidelines for Laboratory Animals Care and Use and approved by the Committee for the Care and Use of Laboratory Animals in the College of Pharmacy, Kyung Hee University.

### Generation of the memory deficient rat model and administration of soya-I

Because senile plaques and neuronal cell death appear in the entorhinal cortex and hippocampus in early AD patients [25], we generated memory deficient model rats by injecting IBO into the entorhinal cortex. The surgery protocol was followed as described with modifications [25-28]. The rats (6 weeks old) were anesthetized with equithensin (350 mM sodium pentobarbital, 250 mM chloral hydrate, 85 mM MgSO_4_, 40% propylene glycol in 10% ethanol, 2 ml·kg^-1^). To induce cell death in the dentate gyrus and hippocampus in addition to the entorhinal cortex, IBO (1.5 μl per animal, 1 mg·ml^-1^) was injected into the entorhinal cortex as follows (IBO group): rats were placed in a Stereotaxic device (Stoelting Co., Wood Dale, IL, U.S.A.) with the incisor bar 3.4 mm below the interaural line. The needle was positioned 10° right to the midsaggital plane. IBO was injected at 3 locations within the entorhinal cortex and medial entorhinal cortex, 0.35 μl per min speed (first, AP: −8.4 mm, ML: −4.8 mm, DV: −4.6 to −4.8 mm; second, AP: −8.4 mm, ML: −4.8 mm, DV: −2.6 to −2.8 mm; third, AP: −8.8 mm, ML: −3.65 mm, DV: −4.8 mm)[25,27,29]. As a sham group, saline was injected at the same locations instead of IBO (sham group). Oral administration of vehicle (2 % Tween 80) without soya-Ι was given to both the sham and IBO groups for 1 week. The sham and IBO groups were used as positive and negative control groups, respectively. Vehicle or soya-I (1 ml once a day) was administered orally by an intubation using oral feeding needle (oral zonde needle, 9cm) and vomiting reflex was not observed. The rats (8 weeks old) were examined for behavioral tests (for about 1 week) at 1 week after oral administration of soya-I (5, 10, and 20 mg·kg^-1^) or vehicle. To examine the effects of proliferation and differentiation of new born NPCs on memory behavior and neuro-regeneration, the rats (12 weeks old) were examined for behavioral tests (in the sequence of Y maze task, passive avoidance task, and then Morris water maze task) at 4 weeks after oral administration of soya-I and then sacrificed for immunohistochemical analysis.

### Y-Maze task

The Y-maze test was performed first among the behavior tests, as previously described [26,30]. The maze was made of black-colored acryl and positioned at equal angles. Rats (8 or 12 weeks old) were separated by groups (Sham n=12, IBO n=12, Soya-1 n=5-6), and then they were habituated in the Y maze recording room for 30 min. The rats were placed at the end of the arm and allowed to move freely through the maze and to enter as many arms as they like during the 8-min sessions. Arm entry sessions were recorded when the hind paws of the rats were completely placed in the arm. Consecutive entry into three arms in an alternative order was defined as successive entries on overlapping triplet sets, and alternation percentage was calculated as the ratio of actual to possible alternations (defined as the total number of arm entries minus 2), multiplied by 100. 

### Passive avoidance task

 Next, the passive avoidance test was carried out as previously described, with the same animal numbers for each group as the Y-maze test [26,31]. The task consisted of a semi-automated system with a shuttle chamber. The rats were trained to avoid the light by entering the dark chamber through the acryl door when the light was turned on. This trail was repeated three times a day until the rat had entered the dark chamber within 20 seconds (training trial) for 3-4 days (instead of a set number of trials). In this study, all rats were successfully trained to enter within 20 seconds on the final training day. After performing the acquisition trial on the last day of training (the acquisition trial), the rats were placed in the lightened chamber and when they entered the dark chamber, the door was closed manually and an electrical foot shock (1 mA) was delivered for 3 seconds through the grid floor. Exactly 24 hours after the acquisition trial, the rats were again placed in the lightened chamber, and the latency time to enter the dark chamber was measured for 720 seconds (the retention trial).

### Morris water maze task

Lastly, the Morris water maze test was conducted (with 13 weeks old rats, Sham n=13, IBO n=13, Soya-1 n=6) in a circular stainless pool (160 cm in diameter; 60 cm in height) with a white-painted inner surface. The pool was filled to a depth of 50 cm with water (maintained at 23.0 ± 1.0 °C). An invisible platform (15 cm circular white) was submerged 1.0 cm below the water surface and placed in the center of the northeast quadrant. Each rat was given one trial per day for 4 days to find the hidden platform (Training trial). A trial was initiated by placing a rat in the water facing the pool wall, in one of the four quadrants randomly, but all four quadrants were used once every day. For each trial, the rat was allowed to swim a maximum of 60 seconds to find the platform and to rest for one minute on the platform upon success. The average times of the four quadrant tests were defined as the escape latency time per group on training days. On the last day of the training trial, the rats were subjected to a probe trial in which the platform was removed from the pool, allowing them to swim for 60 seconds to search for the removed probe position. All swimming times and lengths of the trials were recorded and monitored via a video camera. Tracking was accomplished by following the trajectory of the white rat, which was indicated by a black point against the white background. Captured video pictures were analyzed by a video tracking system (Ethovision water maze program, Noldus information technology, Wageningen, The Netherlands). Analyzed information included swimming time in the target quadrant (the swimming time spent to find the hidden platform in the target quadrant, where the hidden platform was positioned,) and the number of virtual platform crossings frequency to find the removed platform.

### Immunohistochemical assay

Immunostaining analysis of brain slices was carried out as previously described with modifications [26,29]. Rats were transcardially perfused with 4 % PFA in PBS. Following immersion fixed with 4% PFA in PBS for 4 hours, the brains were cryoprotected in 30 % sucrose–PBS and then frozen with optimal cutting temperature (OCT) compound and stored at −80 °C until processed. Brain tissue blocks were cryosectioned through the coronal plane at a thickness of 35 μm. The sections were stored at 4 °C in the storing solution (30 % glycerol, 30 % ethylene glycol in PBS). The cryosectioned brain slices were permeabilized in 0.5 % Triton X-100 for 20 minutes and blocked in 15 % normal serum with 3 % bovine serum albumin (BSA, bio-WORLD, Dublin, OH, USA) and 0.1% Triton X-100 for 2 hours in a free floating condition. The sections were incubated for 16 hours at 4 °C with antibodies against vesicular glutamate transporter1 (VGluT1, dilution ratio 1:500, Chemicon), choline acetyltransferase (ChAT, 1:500, Chemicon), glutamic acid dehydrogenase65/67 (GAD65/67, 1:500, Chemicon), glial fibrillary acidic protein (GFAP, 1:1,000, Chemicon), tyrosine hydroxylase (TH, 1:500, Chemicon), and CD11b/c (OX42, 1:250, Serotec). Secondary antibodies conjugated with Alexa Fluor 488 (1:1,000, Invitrogen), Cy2 (1:500, Jackson) and Cy3 (1:500, Jackson) were used. Nuclei were counterstained with 1 ug·ml^-1^ propidium iodide (PI, Sigma) for 5 minutes. Immunostained sections were scanned with a confocal laser microscope (LSM510, Carl Zeiss, Oberkochen, Germany). Over five animals from each group were used for immunohistochemical analysis. For immunostaining assay of anti-bromodeoxyuridine (BrdU, Sigma) and double staining of BrdU/subtype cell markers, incubation of 2 N HCl at 37 °C for 30 minutes was added after permeabilizing in 0.5 % Triton X-100 for 20 minutes.

### Neural precursor cell cultures from embryonic hippocampus

Time-pregnant SD rats (Orient Animal Breeding Center, LTD., a branch of Charles River Laboratories, Gyunggi-do, Korea) were sacrificed by exposure to CO_2_ inhalation. Embryonic neurons were prepared from E16 rat hippocampi as described previously [32] with slight modifications. Briefly, hippocampi were dissected from the embryonic forebrain aseptically, dissociated mechanically in Ca^2+^/Mg^2+^-free Hank’s balanced salt solution and then plated at 7×10^5^ cells/cm^2^ on 60 mm dishes pre-coated with 15 ug·ml^-1^ poly-L-ornithine and 1 ug·ml^-1^ fibronectin. NPCs were cultured in serum-free N2 media supplemented with 10 ng/ml bFGF in 5 % CO_2_ for 3 days before passaging using 0.05 % trypsin-ethylenediaminetetraacetic acid and then grown in the same media for an additional day. For immunoblot assays of VGluT1, ChAT, and GAD65/67, they were grown on a 60 mm dish for 6 more days for differentiation without bFGF and with vehicle (vehicle group) or soya-Ι (0.5, 1, and 2 μM; soya Ι-treated group). For immunocytochemical assays of proliferation using Ki67 antibodies, they were grown on 12 mm glass coverslips for 1 more day without bFGF and with vehicle (dimethyl sulfoxide, vehicle group) or soya-Ι (0.5, 1, and 2 μM; soya Ι-treated group). For immunocytochemical assays of neuronal differentiation, they were grown on coverslips for 4 more days (NeuN) or 2 more days (double-immunostaining of GFAP and NeuN, and TUJ1 and MAP2) without bFGF and with soya-Ι (0.5, and 1 μM).

### Immunocytochemical staining assay

Immunostaining of neuronal precursor cells (NPCs) was carried out as previously described with modifications [29]. Hippocampal precursor cells were cultivated in 12 mm glass coverslips (Bellco Co., Vineland, NJ, USA) at 2×10^4^ cells/well for 1 day, and then the medium was replaced with N2 medium without FGF. On the next day, soya-Ι at three different concentrations, 0.5, 1, and 2 uM were added into the medium for another day, and then the cells were fixed with 4% PFA in PBS for 15 minutes at 4 °C. The cells were permeabilized with 0.5 % Triton X-100 for 5 minutes and blocked with 5 % normal serum (the mixture of normal donkey, goat, and horse serum) for 1 hour at room temperature. Primary antibodies were incubated for 1 hour at room temperature with anti-Ki67 (1:500, Abcam), anti-GFAP (1:1,000, Chemicon), anti-NeuN (1:500, Chemicon), anti-MAP2 (1:2,500, Sigma), and anti-TUJ1 (1:2,000, Sigma) antibodies. Secondary antibodies conjugated with Cy3, Cy5 (1:700, Jackson) or Alexa 488 (1:1,000, Invitrogen) were used. Nuclei were counterstained with 1 μg·ml^-1^ PI for 5 minutes. Immunostained cells were scanned under a confocal laser microscope (LSM10, Carl Zeiss).

### Immunoblotting assay

Hippocampal precursor cells were lysed in 80 μl of ice-cold RIPA buffer containing a protease inhibitor cocktail. After centrifugation for 10 minutes at 13,000×g, the supernatants were divided into Eppendorf tubes and stored at − 80 °C. Protein quantification assay was performed using Bradford protein assay kits (Bio-Rad, Hercules, California, USA). For immunoblot analysis, a mix of sample loading buffer (Biosesang Co., Seoul, Korea) and 20 μg of protein were boiled at 100 °C for 10 minutes. Denatured proteins were separated by 10 % polyacrylamide gel electrophoresis for 2–3 hours at 100 V, and transferred to a 0.2 μm nitrocellulose membrane for 2-3 h at 100 V. Membranes were then washed for 15 minutes 3 times in 0.1% Tween-20 PBS between each of following steps: 1 hour block in 5 % milk, over-night incubation at 4 °C with primary antibodies against -VGluT1 (1:400, Chemicon), GAD65/67 (1:500, Chemicon), ChAT (1:400, Chemicon), and β-actin (1:1,000, SantaCruz, Delaware, California, USA), and 1 hour incubation at room temperature with secondary antibodies. 

### Quantification and statistics

Cell counting was performed as reported by [33]. The average length per neurite of MAP2-positive cells with longer neurites than double the cell body width was counted from 10~12 confocal microscopic fields that were randomly chosen per cultured cover slip. Primary branches, which directly outgrow from the cell body, were counted when the length of primary branches was more than 2-fold of cell body width. All secondary and tertiarybranches that come from primary and secondary branches, respectively, were counted. The numbers of the branches were also counted from 10~12 confocal microscopic fields randomly chosen from each cover slip that neuronal cells were grown on and immunostained with MAP2 antibodies. Analysis of neurite length and numbers of branches was performed with LSM image examiner ver. 2.80 (Carl Zeiss).

In the immunohistochemical analysis, the number of immunostained cells against specific antibodies was counted in confocal images of five or six hippocampal coronal-sections. For each staining analysis, every fifth cryosections of the hippocampal region of brain tissues (AP: Bregma -4.5 to -4.3 mm) were taken to immunostain. The number of immunostained cells was presented as an average of cell numbers in the hippocampal field of one brain slice. In brain slice samples one week and four weeks after soya-I administration, VGluT1 and GAD-positive cells were presented as cell numbers per microscophic field (x400-VGluT1, x800-GAD67) and ChAT-positive cells per each hippocampal field (10-12 confocal microscopic fields) of brain slices. Five to eleven animals per group were used. Western blotted membranes were analyzed using the Multi-gauge, bio-imaging program on the LAS-4000 mini (Fujifilm Lifescience, Stamford, CT, USA). Densitometric analyses of the expression ratios of VGluT1/β-actin, GAD65/67/β-actin, and ChAT/β-actin were normalized to the vehicle group. All the behavioral data, cell count data and densitometric data were expressed as the mean ± SEM, and statistical significance was analyzed by one-way analysis of variance (ANOVA) followed by the Newman-Keuls Multiple Comparison Test or unpaired *t* test (Graph Pad Prism, version 5.01). A Power analysis was conducted using G*Power version 3.1.7 [34]. Statistical significance was set at *p*<0.05.

## Results

### Behavioral effects of soya-Ι on learning and memory impairment in rats induced with ibotenic acid

To test whether soya-Ι has effects on recovery from memory impairment, we administered soya-I orally to memory-deficient model rats. The model rats were generated by stereotaxic microinjection of ibotenic acid (IBO) into the entorhinal cortex of adult male rats as described previously [25-28,35,36]. In the model animals, injection of IBO induced cell death in the CA regions and the DG of the hippocampus, as well as the entorhinal cortex, along with loss of hippocampus-related learning and memory abilities. 

**Figure 1:**
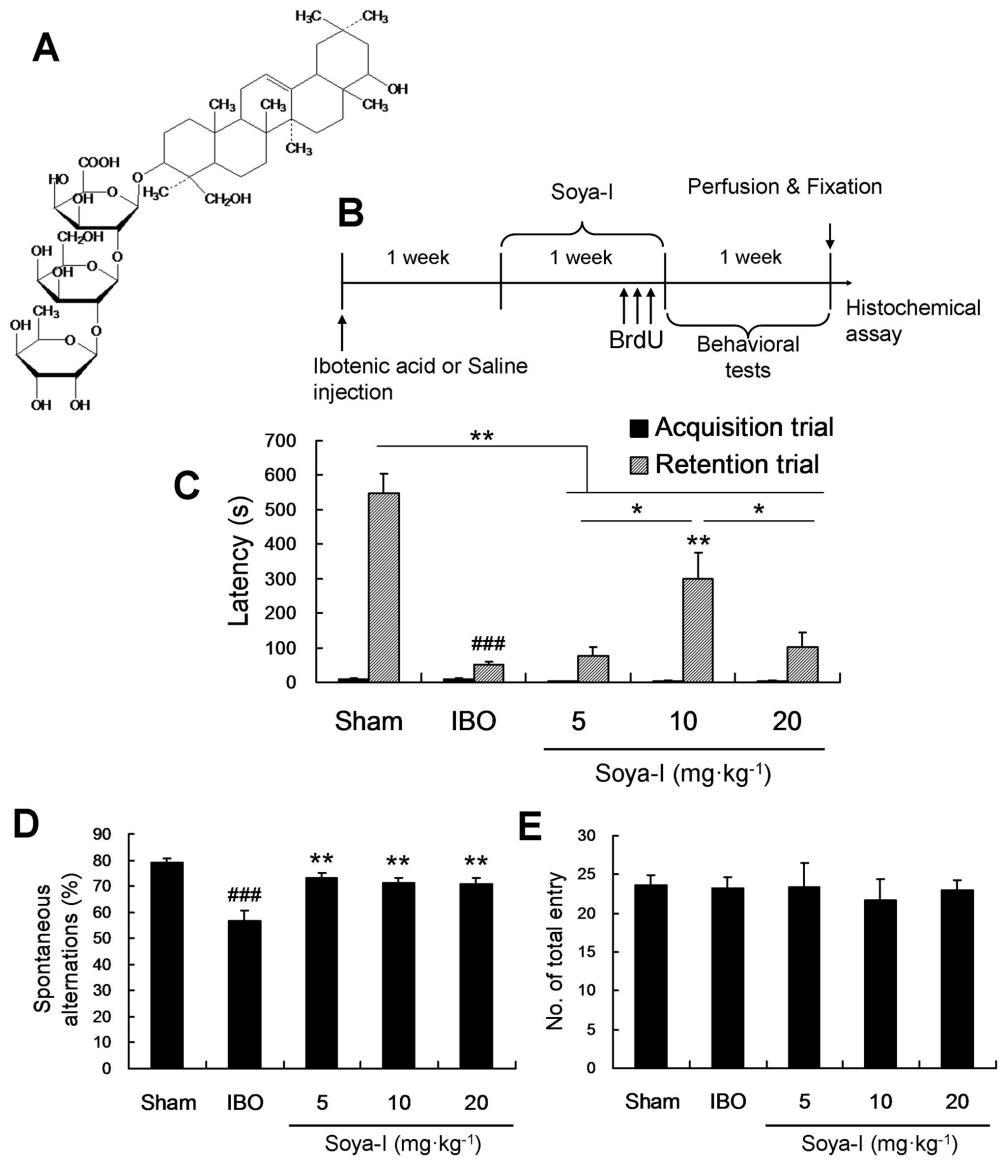
Effects of soyasaponin Ι (soya-Ι) on memory behaviors in ibotenic acid induced model rats. A. Structure of soya-I isolated from soybean (*Glycine Max* Marr., family: Leguminosae), B. time line of experimental procedures for the investigation of proliferation and differentiation, and behavioral effects of soya-I. Rats were orally administered soya-Ι (5, 10, and 20 mg·kg^-1^, p.o.) or vehicle (same volume, p.o.) once per day for 7 days, and the passive avoidance tests (C) and Y-maze tests (D) were carried out 1 week later. Rats were orally administered soya-Ι (10 mg·kg^-1^, p.o.) or vehicle (same volume, p.o.) once per day for 7 days. The sham group was injected with saline instead of IBO. C. Latency times in the passive avoidance test, D. spontaneous alterations, and E. number of total entry in the Y-maze test during 8-min sessions were measured as described in Materials and Methods. Data represent means ± standard error of the mean (SEM). ^###^ *p* < 0.001, compared with the sham group; ^*^ *p* < 0.05, ^**^ *p* < 0.01, compared with the IBO group by the Newman-Keuls Multiple Comparison Test.

We isolated soya-Ι with more than 95% purity as reported previously [22]. The chemical structure of soya-I is shown in Figure 1A. We orally administered soya-Ι at 5, 10, and 20 mg·kg^-1^ once daily for 1 week to memory-impaired rats induced with IBO. After administration of soya-Ι, we conducted behavioral tests including the Y-maze and passive avoidance tests (Figure 1B).

In the passive avoidance tests, rats were trained for 4 days until entering the dark chamber within 20 seconds. In the acquisition trial, which carried out on the last day of the training (the acquisition trial), latency times of all groups were not significantly different (Figure 1C). Twenty four hours after the rats received a shock, we measured the latency time for the rats to enter the dark. In the retention trial, the saline-injected sham group did not enter the dark room for up to 547 s (latency time). In contrast, the latency time of the memory-deficient rat group injected with IBO (IBO group) was markedly reduced (### p < 0.001 by Newman-Keuls Multiple Comparison Test, IBO group compared with Sham group). However, the memory-deficient rat group treated with 10 mg·kg^-1^ soya-I (soya-I group) showed significantly increased latency times compared with the IBO group (*p < 0.05 by Newman-Keuls Multiple Comparison Test). In the 10 mg·kg^-1^ soya-I-treated group, the latency time increased to the highest level, 54.5 % of the latency time of the sham group (F_4,34_ = 24.32, p < 0.0001 by One-way ANOVA; Figure 1C). 

Next, in the Y‑maze task showing spatial memory, rats faced a choice in selecting a pathway in the Y‑shaped track. Spontaneous alterations in arm entries were lower in the IBO group compared with the saline-injected sham group (### p < 0.001 by the Newman-Keuls Multiple Comparison Test). However, the reduction in memory observed in the IBO-treated rats was improved considerably by oral administration of soya-Ι (** p < 0.01 by Newman-Keuls Multiple Comparison Test; F_4,34_ = 10.64, p < 0.0001 by One-way ANOVA; Figure 1D). The number of arm entries was not substantially different across all groups (Figure 1E), indicating that alterations in behavior are not caused by increased movements or environmental changes. 

These behavioral results suggest that oral administration of soya-Ι in memory-deficient model rats improve their learning and memory abilities.

### Soya-Ι increased regeneration of neuronal cells in the hippocampus of IBO-treated rats

**Figure 2:**
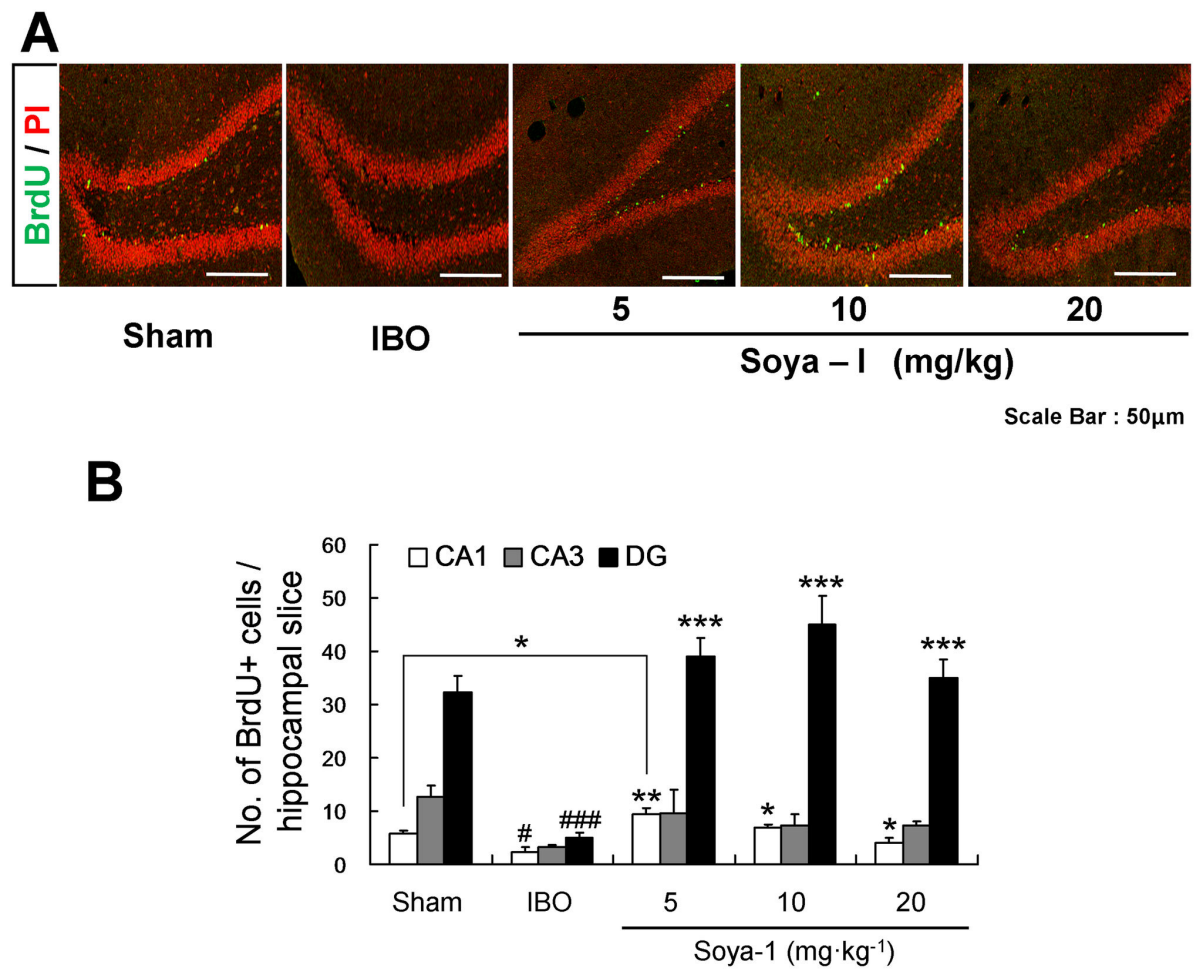
Proliferation of Neural precursor cells increased by oral administration of soya-Ι in memory-deficient rats. A. Confocal laser scanning microscopic images of neuronal cells in the hippocampal region of brain slices, immunostained with a BrdU marker (green: BrdU-positive cells, red: propidium iodide (PI)). B. The average numbers of BrdU-positive cells in the hippocampal region per brain slice. Five different animals were used for each treatment group. Data represent means ± SEM (^#^ *p* < 0.05, ^###^ *p* < 0.001, compared with the sham group, ^*^ *p* < 0.05, ^**^ *p* < 0.01, ^***^ *p* < 0.001, compared with the IBO group by the Newman-Keuls Multiple Comparison Test).

**Figure 3:**
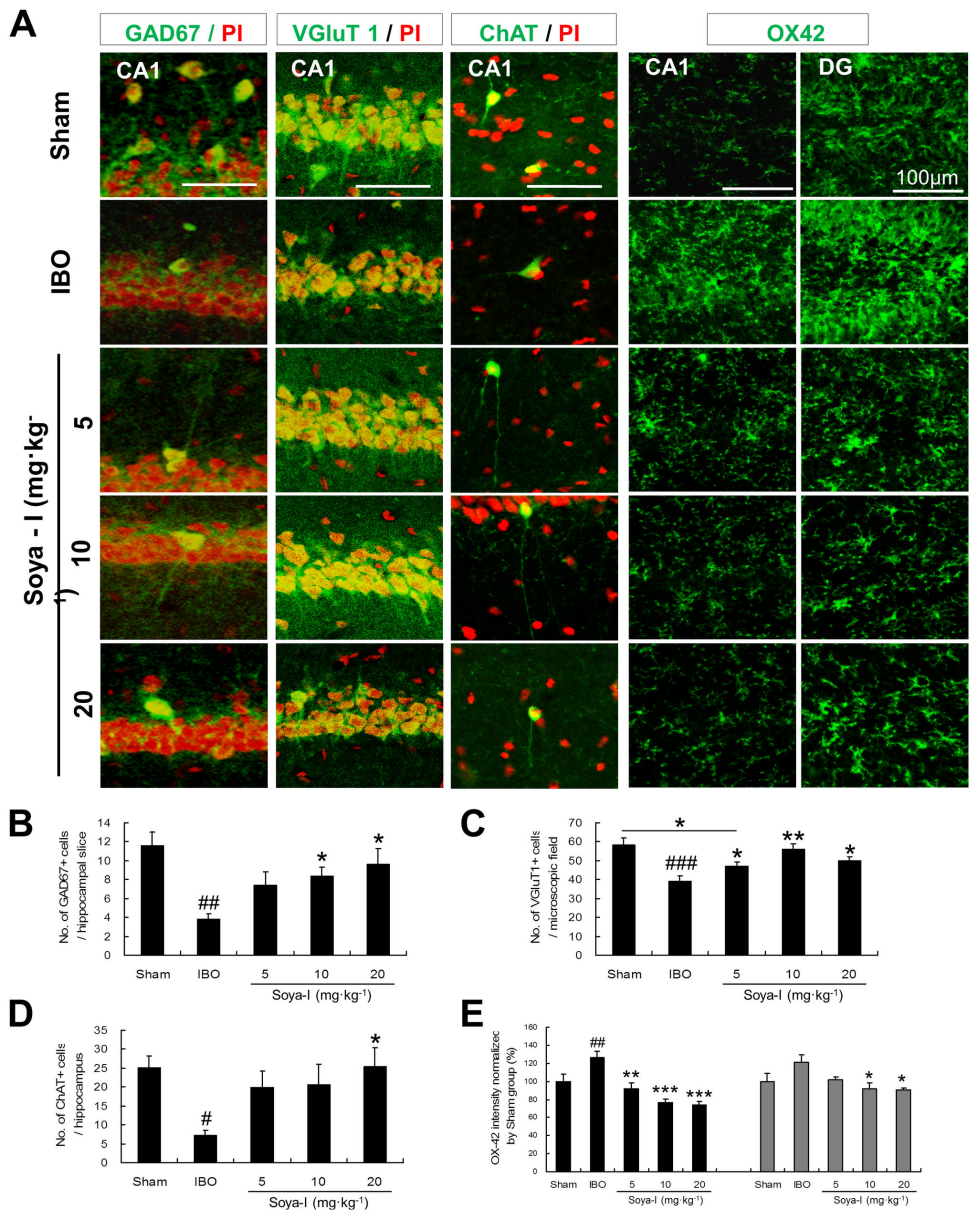
Neuronal cell types and microglia activation affected by soya-Ι administration in memory-deficient rats. A. Confocal laser scanning microscopic images of GAD67, VGluT1, ChAT, OX42-positive cells in the hippocampal region of brain slices (green: marker-positive cells, red: propidium iodide (PI)). B. The numbers of GAD67-positive cells in the hippocampal region of rat brain slices (soya-I-treated and IBO-injected control) compared with the saline-injected sham group. C. The numbers of VGluT1-positive cells in a microscopic field of the CA1 region in the hippocampal region of IBO-injected rat brain slice compared with the sham group. D. The numbers of ChAT-positive cells in the hippocampal region of IBO-injected rat brain slices, compared with the sham group. E. The average intensities of OX42-positive cells in a microscopic field of the CA1 and DG were normalized according to the sham group. The numbers of PI-positive cells were between 80 and 110 cells in a microscopic field. Immunohistochemical analyses were conducted as described in Materials and Methods (intensity of OX42 staining was analyzed using the ImageJ software: http://rsb.info.nih.gov/ij/). Five different animals were used for each treatment group. Data represent means ± SEM (^#^ *p* < 0.05, ^##^ *p* < 0.01, ^###^ *p* < 0.001, compared with the sham group, ^*^ *p* < 0.05, ^**^ *p* < 0.01, ^***^ *p* < 0.001 compared with the IBO group by the Newman-Keuls Multiple Comparison Test).

Based on the animal behavior test results, we investigated how soya-Ι facilitates the learning and memory abilities of memory deficient model rats. To examine whether soya-I could facilitate the proliferation and differentiation of hippocampal cells, which are responsible for the formation of learning and memory, we performed immunohistochemical staining of rat brain tissues using markers for cell proliferation (BrdU) (Figure 2) and neuronal subtypes (ChAT, vGluT1, and GAD67) (Figure 3). Immunostaining was visualized using secondary antibodies conjugated with fluorescence dye and scanned using a confocal laser scanning microscope. The number of endogenous BrdU-positive cells in the hippocampal region was increased by oral administration of soya-Ι (Figure 2A), compared with the IBO group. In particular, the number of BrdU-positive cells in the DG showed a significant increase with all doses (5, 10, and 20 mg·kg^-1^ groups) of orally administered soya-Ι. Soya-I at 10 mg·kg^-1^ resulted in the largest increase in the number of BrdU-positive cells (Sham n = 4, IBO n = 3, Soya-I 5 mg·kg^-1^ n = 3, Soya-I 10 mg·kg^-1^ n = 3, Soya-I 20 mg·kg^-1^ n = 3; F_4,11_ = 18.18, p < 0.0001 by One-way ANOVA; Figure 2B). 

Next, we performed immunohistochemical staining to detect differentiation markers for neuronal subtypes which mainly contribute to learning and memory in the hippocampus and entorhinal cortex (Figure 3). In the entorhinal cortex of IBO-injected rats (IBO), we observed that neuronal cell types expressing specific markers such as VGluT1 (glutamatergic neurons), GAD67 (GABAergic neurons), and ChAT (cholinergic neurons) were reduced by 55 %, 50 %, and 80 %, respectively, compared with saline-injected rats (sham; Figure S1). In the hippocampus of IBO-injected rats, the number of VGluT1-positive cells decreased, to 72.44 ± 3.86 %, compared with the sham group (100 ± 6.50 %). In addition, GAD67-positive cells (43.02 ± 3.72 %) and ChAT-positive cells (52.07 ± 7.38 %) decreased, compared with the sham group (GAD67: 100±11.43, ChAT: 100±1.64; Figure 3A-D). This indicates that injecting IBO into the entorhinal cortex, where many neurons project to the hippocampus, decreases the numbers of major neuronal cell types that participate in the formation of hippocampal memory, such as glutamatergic, GABAergic, and cholinergic neurons. 

At the three doses tested (5, 10, and 20 mg·kg^-1^), soya-I increased GAD67-positive cells in the hippocampal region approximately 1.66- to 1.76-fold (6.83 ± 1.47 cells to 7.86 ± 1.62 cells per hippocampal slice), in comparison with the IBO group (3.81 ± 0.63 cells, Sham n = 5, IBO n = 5, Soya-I 5 mg·kg^-1^ n = 5, Soya-I 10 mg·kg^-1^ n = 5, Soya-I 20 mg·kg^-1^ n = 5; F_4,20_ = 5.191, p = 0.0049 by One-way ANOVA; Figure 3A, B), in a dose-dependent manner, and VGluT1-positive cells were slightly increased up to the level of the sham group (57.33 ± 2.43 cells per microscopic field at 10 mg·kg^-1^) in comparison with 41.00 ± 2.88 cells in the IBO group (Sham n = 5, IBO n = 5, Soya-I 5 mg·kg^-1^ n = 5, Soya-I 10 mg·kg^-1^ n = 5, Soya-I 20 mg·kg^-1^ n = 5; F_4,20_ = 8.127, p = 0.0005 by One-way ANOVA; Figure 3C). In addition, ChAT-positive cells in the hippocampal region were elevated 1.39- to 2.23-fold (17.64 ± 4.23 cells to 20.93 ± 4.50 cells) compared with the IBO group (7.30 ± 1.32 cells; Sham n = 5, IBO n = 5, Soya-I 5 mg·kg^-1^ n = 5, Soya-I 10 mg·kg^-1^ n = 5, Soya-I 20 mg·kg^-1^ n = 5; F_4,20_ = 3.314, p = 0.0308 by One-way ANOVA; Figure 3D). These results indicate that soya-I promotes proliferation of NPCs and survival of differentiated neuronal cells such as GABAergic, glutamatergic, and cholinergic neurons, and thereby facilitates neuronal regeneration. 

Next, we assessed whether soya-I affects neuroinflammation. We conducted immunohistochemical staining of rat brain tissues using a marker for reactive microglia (OX42). As the intensity of OX42-positive cells in the DG and CA1 regions in the IBO group was normalized to the intensity of the sham group, the normalized intensity in the IBO group showed a significant increase compared with the sham group (Figure 3E). However, the normalized intensity was decreased in the soya-Ι-treated groups in a dose-dependent manner in both the CA1 and DG regions (Sham n = 5, IBO n = 5, Soya-I 5 mg·kg^-1^ n = 5, Soya-I 10 mg·kg^-1^ n = 6, Soya-I 20 mg·kg^-1^ n = 5; CA1, F_4,21_ = 12.83, p < 0.0001 by One-way ANOVA; DG, F_4,21_ = 3.559, p = 0.0229 by One-way ANOVA). Taken together, these data suggest that soya-I has multi-actions affecting neuronal regeneration and protection by inhibiting degeneration and inflammation induced by IBO treatment. 

### Soya-Ι supported maintenance of memory recovery and neuronal regeneration in the hippocampus

**Figure 4:**
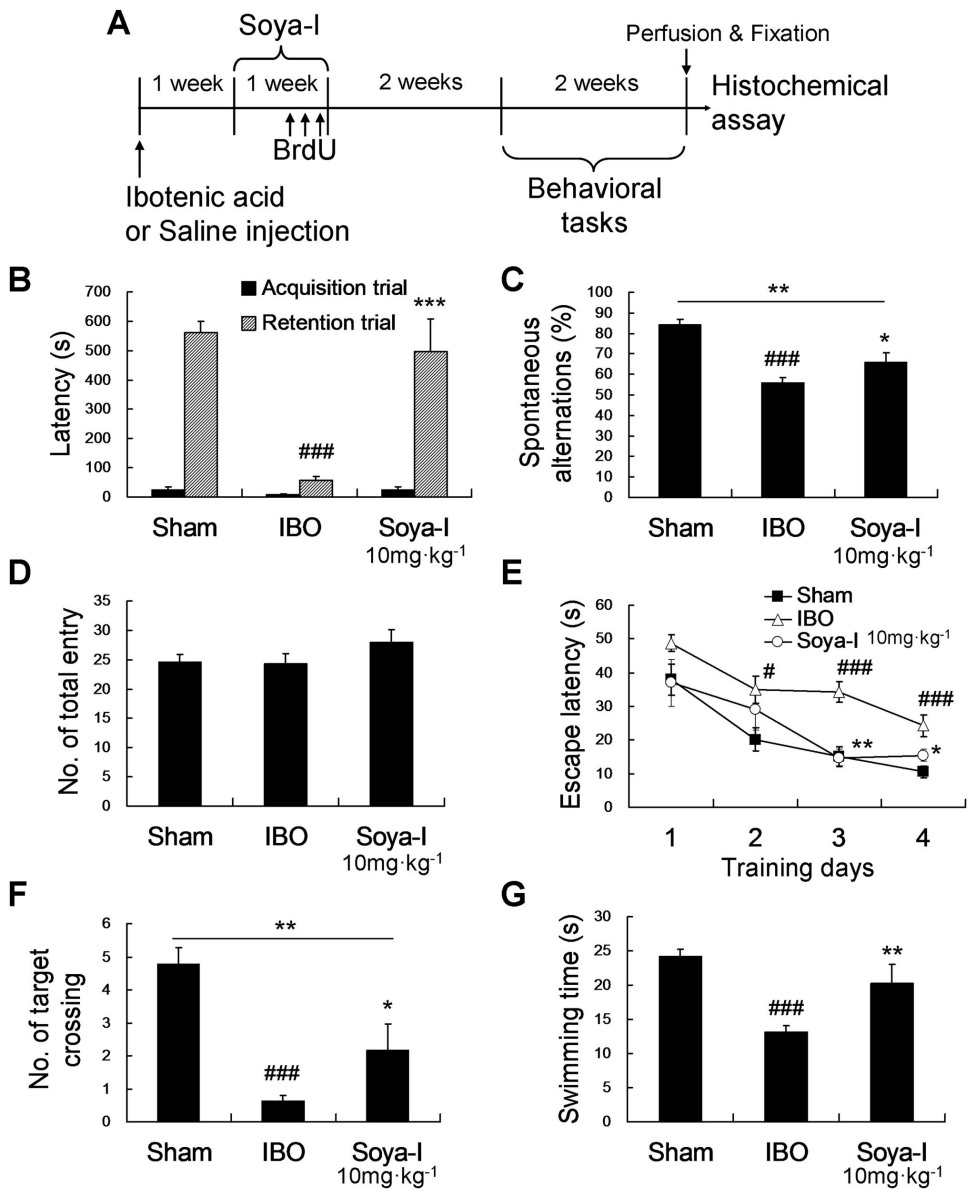
Effects of soya-Ι on behavioral tests 4 weeks after soya-I administration in memory-deficient rats. A. A time line of experimental procedures B. Behavioral effect of soya-I on memory-deficient model rats 4 weeks after oral administration. Rats were orally administered soya-Ι (10 mg·kg^-1^, p.o.) or vehicle (same volume, p.o.) once per day for 7 days, and passive avoidance tests (B) and Y-maze tests (C) were carried out 3 weeks later. The sham group was injected with saline instead of IBO. B. Latency times in passive avoidance test and C. spontaneous alterations in the Y-maze test during 8-min sessions were measured as described in Materials and Methods. Rats were orally administered soya-Ι (10 mg·kg^-1^, p.o.) or vehicle (same volume, p.o.) once per day for 7 days, and the Morris water maze task was carried out 3 weeks later. The sham group was injected with saline instead of IBO. D. Total entries were not significantly different. E. The mean escape latency to find the hidden platform during 4 consecutive days of training trials, F. the number of virtual platform crossings and G. swimming time in the target quadrant in 60-s probe trials with no platform were measured as described in Materials and Methods. Closed square = Sham group, opened triangle = IBO group, open circle = soya-I treated group. Data represent means ± SEM (^#^ *p* < 0.05, ^##^ *p* < 0.01, ^###^ *p* < 0.001, compared with the sham group, ^*^ *p* < 0.05, ^**^ *p* < 0.01, ^***^ *p* < 0.001, compared with the IBO group by the Newman-Keuls Multiple Comparison Test).

 If newly born NPCs survive and get incorporated into the neural network in addition to neuronal cell types increased at 1 week, abilities of memory formation could be maintained. We investigated whether the effect of soya-I on memory recovery at 1 week after the administration could be maintained over 4 weeks by conducting the Y-maze, passive avoidance, Morris water maze tests. The behavioral tests were performed 4 weeks after oral administration of soya-Ι at 10 mg·kg^-1^ once daily for 1 week (Figure 4A), because memory recovery was best at 10 mg·kg^-1^ soya-I (Figure 1B). In the passive avoidance test, the latency time of the soya-Ι-treated group was recovered dramatically compared with IBO group and to 89.65 % of that of the sham group (^###^ p < 0.001 by Newman-Keuls Multiple Comparison Test, IBO group were compared with Sham group, ^***^ p < 0.001 by Newman-Keuls Multiple Comparison Test, Soya-I 10 mg·kg^-1^ group were compared with IBO group; F_2,27_ = 40.57, p < 0.0001 by One-way ANOVA; Figure 4B). In the Y-maze test, spontaneous alterations in the soya-Ι-treated group showed a significant increase, by 78.57 %, compared with the IBO group (^###^ p < 0.001 by Newman-Keuls Multiple Comparison Test, IBO group were compared with Sham group, ^**^ p < 0.01 by Newman-Keuls Multiple Comparison Test, Soya-I 10 mg·kg^-1^ group were compared with IBO group; F_2,27_ = 25.57, p < 0.0001 by One-way ANOVA; Figure 4C), whereas the numbers of total entries were not significantly different among all groups (Figure 4D). In the training trial session of the Morris water maze test, the escape latency time for finding the hidden platform in the sham and soya-Ι groups declined progressively during the training period of 4 consecutive days in comparison with the IBO group. In particular, on the third and fourth days, the escape latency time in the soya-Ι-treated group was reduced than that of the IBO group (Day 3, F_2,29_ = 14.29, p < 0.0001; Day 4, F_2,29_ = 9.985, p = 0.0004; Figure 4E). In the probe trial session, the number of crossings across the target probe and swimming times within the target quadrant in the soya-Ι-treated group showed reduced tendency in the IBO group and recovered almost that of the sham group (Swimming time, F_2,29_ = 21.07, p < 0.0001; target crossing, F_2,29_ = 25.63, p < 0.0001; Figure 4F, G). These results indicate that the memory improvements in behavioral tests resulting from soya-I administration lasted for 4 weeks after the administration.

**Figure 5:**
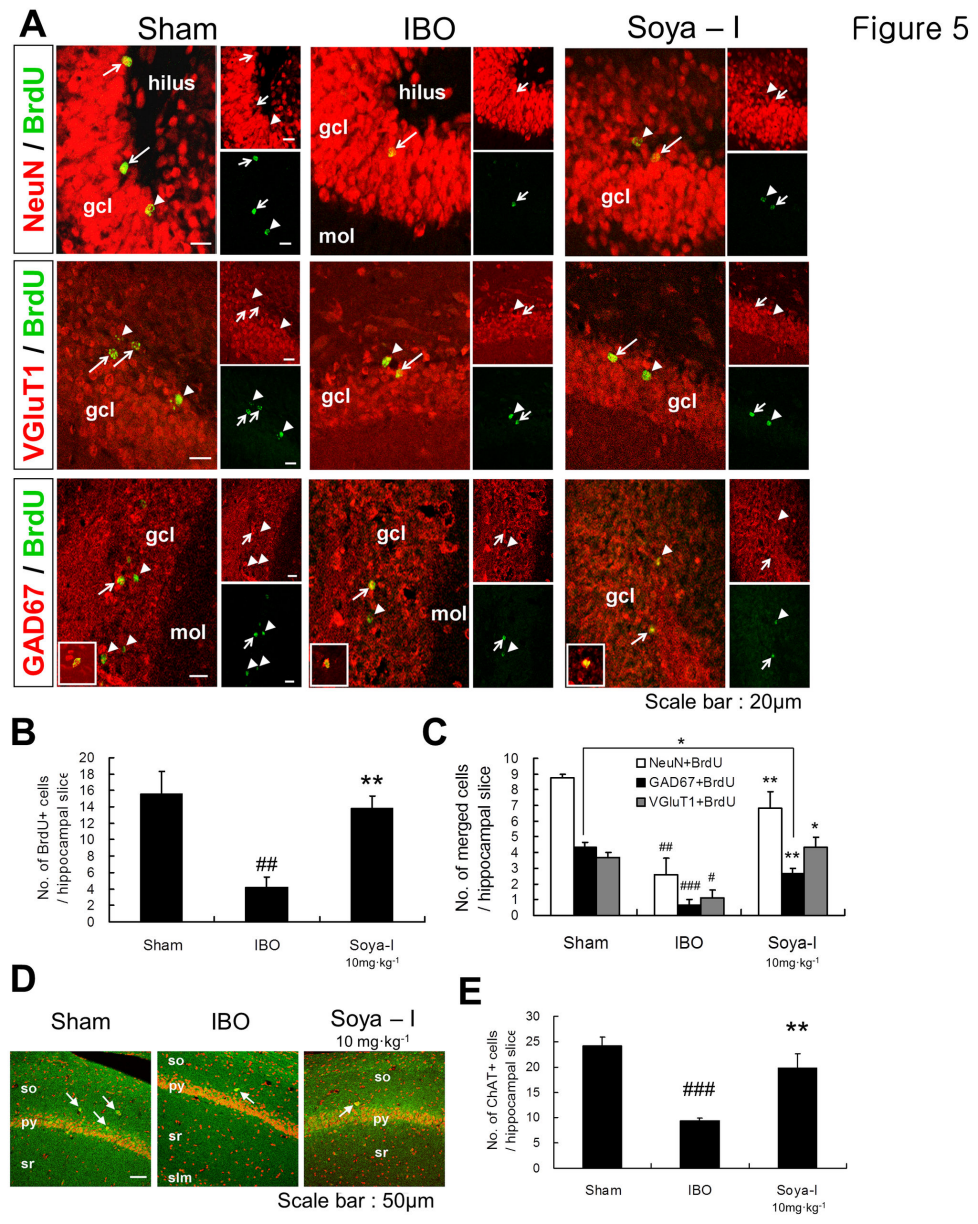
Differentiation of new-born NPCs to neuronal cell types induced by soya-Ι administration. A. Confocal laser scanning microscopic images of neuronal cells merged with isotype cell markers in the granular cell layer of brain slices (green: BrdU-positive cells, red: VGluT1, GAD67, and NeuN). Arrow heads = non-merged cells, arrows = merged cells (the image below left; BrdU and GAD67-double positive cells). B. Average numbers of BrdU-positive cells per hippocampal slice. C. Average numbers of NeuN-, VGluT1-, and GAD67-positive cells merged with BrdU-positive cells per hippocampal slice. D. Confocal laser scanning microscopic images of ChAT-positive cells (arrows) in the hippocampal region of brain slices (green: ChAT-positive cells, red: PI). Abbreviations: so, stratum oriens; py, pyramidal layer of CA1; sr stratum radiatum; slm, stratum lacunosum moleculare. E. Average numbers of ChAT-positive cells in the hippocampal region per brain slice. Immunohistochemical assay and counting were carried out as described in the Materials and Methods. Five different animals were used for each treatment group. Data represent means ± SEM (^#^ *p* < 0.05, ^##^ *p* < 0.01, ^###^ *p* < 0.001, compared with the sham group, ^*^ *p* < 0.05, ^**^ *p* < 0.01, compared with the IBO group by the Newman-Keuls Multiple Comparison Test).

To investigate whether elevated memory abilities could maintained by survival and differentiation of newly born DGCs increased by soya-I, we estimated that BrdU-positive cells merge with markers for mature neurons and cell subtypes (NeuN, VGluT1, and GAD67) at 4 weeks after oral administration of soya-Ι (Figure 5A, B). The total number of BrdU-positive cells in hippocampal slices at 4 weeks after soya-I administration (Figure 5B) was reduced to less than half compared with that at 1 week after soya-I administration (Figure 2B). In contrast, the number of BrdU-positive and newly born DGCs was approximately 3.5-fold higher in the soya-Ι-treated group, compared with that in the IBO group (Sham n = 5, IBO n = 5, Soya-I 10 mg·kg^-1^ n = 5; F_2,12_ = 9.975, p = 0.0028 by One-way ANOVA), indicating that the survival of newly born cells was maintained for at least 4 weeks. The number of cells immunostained with both NeuN (a mature neuron marker) and BrdU was three times higher in the soya-Ι-treated group compared with that in the IBO group (Sham n = 4, IBO n = 5, Soya-I 10 mg·kg^-1^ n = 5; F_2,11_ = 11.18, p = 0.0022 by One-way ANOVA; Figure 5A, C). The numbers of BrdU-positive cells merged with VGluT1 and GAD67 in the soya-Ι-treated group were 3.5 and 2.8 times higher, respectively, than that in the IBO group (Sham n = 3, IBO n = 3, Soya-I 10 mg·kg^-1^ n = 3; GAD67+BrdU, F_2,6_ = 30.33, p = 0.0007 by One-way ANOVA; VGluT1+BrdU, F_2,6_ = 10.94, p = 0.01 by One-way ANOVA; Figure 5B, C). However, the ratio of VGluT1-, NeuN- and GAD67-positive cells merged with BrdU-positive cells did not show any significant difference (Figure 5B, C). 

Hippocampal cells labeled with markers for astrocytes (GFAP), dopaminergic neurons (TH), and cholinergic neurons (ChAT) and those merged with BrdU-positive cells were barely detected in any group (data not shown), probably because new born cells mostly migrate to granular cell layers consisting of glutamatergic and GABAergic cells. We found that cholinergic neurons, which was increased at 1 week after soya-I administration, were maintained at a similar level (Figure 3G) until 4 weeks after administration (Sham n = 5, IBO n = 5, Soya-I 10 mg·kg^-1^ n = 5; F_2,12_ = 15.78, p = 0.0004 by One-way ANOVA; Figure 5D, E), when total endogenous ChAT-positive cells were immunostained, suggesting that administration of soya-I may neuroprotect cholinergic neurons from degeneration induced by IBO injection. 

Taken together, these results indicate that soya-I support neuronal survival and differentiation of newly born NPCs into glutamatergic and GABAergic neurons. The NPCs differentiated preferentially to neurons rather than astrocytes. The neuronal cell types were glutamatergic and GABAergic cells rather than dopaminergic and cholinergic cells (Figures 3, 5).

### Soya-Ι promotes neurogenesis and neurite outgrowth in NPCs cultured from rat hippocampus

**Figure 6:**
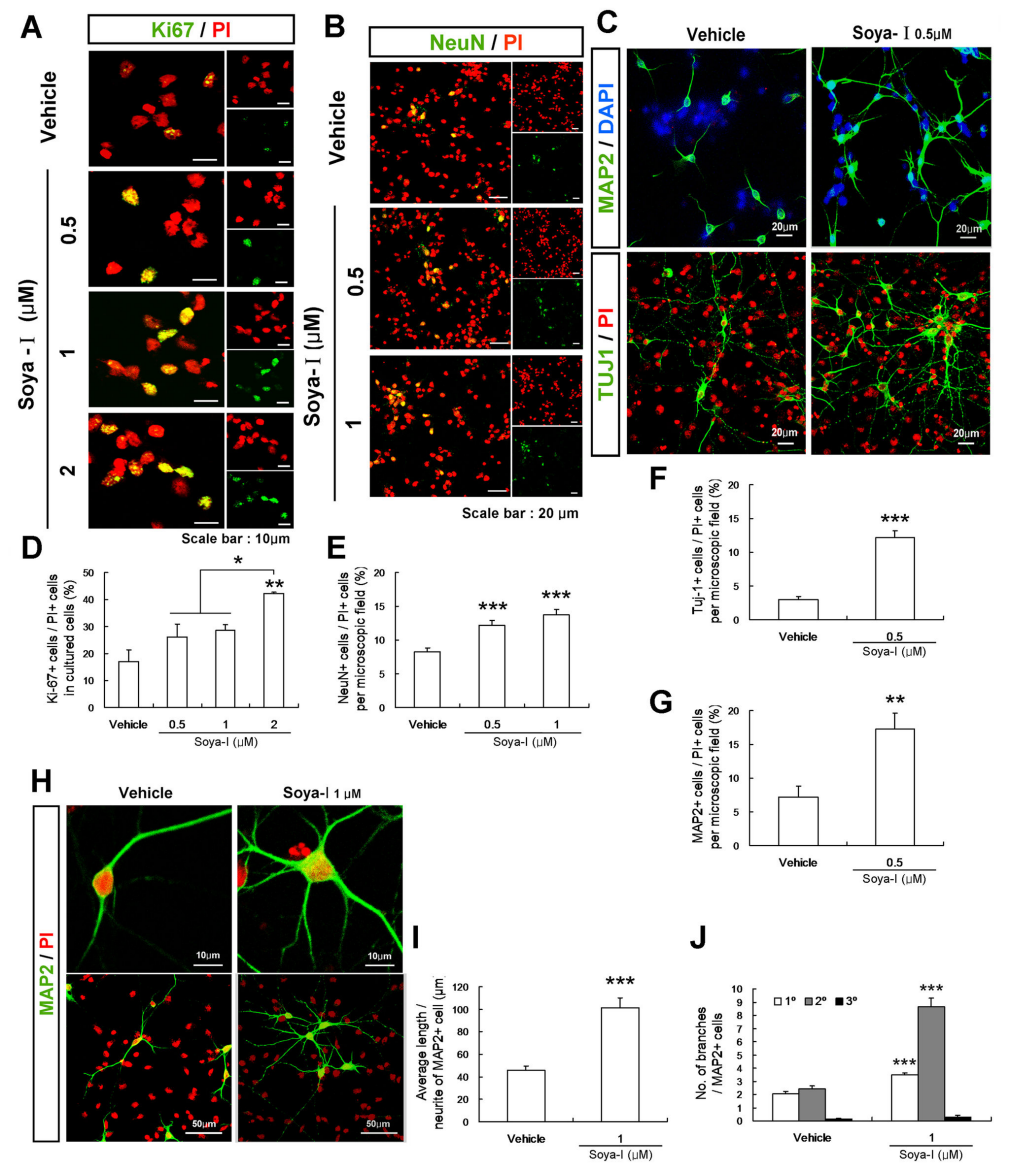
Proliferation and maturation of NPCs and neurite outgrowth of MAP2-positive neurons increased by soya-Ι among cultured hippocampal cells. A. Confocal laser scanning microscopic images of Ki67-positive NPCs (green: Ki67-positive cells, red: PI). B. Confocal laser scanning microscopic images of NeuN-positive cells cultured from the rat embryonic hippocampus (green: NeuN-positive cells, red: PI). C. Confocal laser scanning microscopic images of MAP2 and TUJ1-positive NPCs (green: TUJ1-positive cells and MAP2-positive cells, red: PI). D. Average percentages of Ki67-positive cells, E. Average numbers of NeuN-positive cells, F. Average numbers of TUJ1-positive cells, G. Average numbers of MAP2-positive cells compared with PI-positive cells per microscopic field. H. Confocal laser scanning microscopic images of MAP2-positive cells cultured from the rat embryonic hippocampus (green: MAP2-positive cells, red: PI). I. Average neurite length of MAP2-positive cells with neurites longer than double the cell body width. J. The average number of branches per MAP2-positive cell, where the length of the primary branches is two-fold longer than the width of the cell bodies. All assays and counting were carried out as described in Materials and Methods. Over ten positions per cover slip (three times of Ki67, NeuN, TUJ1, and MAP2 immunostaining assay per one trial hippocampal primary culture carried out) were selected and counted. Data represent means ± SEM. (^*^ *p* <0.05, ^**^ *p* <0.01, ^***^ *p* <0.001, compared with the vehicle group by the Newman-Keuls Multiple Comparison Test (D,E) or unpaired *t* test (F, G, I, J)).

Finally, we investigated whether soya-Ι could directly induce the proliferation or differentiation of NPCs. We conducted immunocytochemical assays in primary NPCs isolated from the embryonic day 16 (E16) rat hippocampus, where pyramidal cell progenitors begin to proliferate. We subcultured the hippocampal cells once to differentiate and immunostain with markers for cell proliferation (Ki67), neuronal differentiation (NeuN, TUJ1), and neurite outgrowth (MAP2). For immunobloting analyses, cell extracts were prepared to detect VGluT1, GAD65/67, and ChAT after 12 days of differentiation. The primary cells in culture were treated with soya-I for the last 1 day for proliferation or 6 days for differentiation. In this analysis, the numbers of cells stained with propidium iodide (PI-positive cells) were usually 80~110 cells in a microscopic field. An increase in Ki67-positive NPCs was observed in all groups treated with soya-Ι (0.5, 1, and 2 μM) for 1 day. Soya-Ι at 2 μM increased the number of proliferating cells (2.5-fold compared with vehicle treatment; Vehicle n = 3, Soya-I 0.5 μM n = 3, Soya-I 1 μM n = 3, Soya-I 2 μM n = 3; F_3,8_ = 9.426, p = 0.0053 by One-way ANOVA; Figure 6A, D). We also observed that addition of soya-Ι at 0.5 and 1 μM into NPCs cultured from the hippocampus promoted differentiation into late-stage NPCs (TUJ1-positive cells; Vehicle n = 8, Soya-I 0.5 μM n = 8; p < 0.0001 by unpaired *t* test; Figure 6C, F) and mature neurons (NeuN-positive cells; Vehicle n = 12, Soya-I 0.5 μM n = 12, Soya-I 1 μM n = 14; F_2,35_ = 14.21, p < 0.0001 by One-way ANOVA; Figure 6B, E) as well as neurite-growing cells (MAP2-positive cells; Vehicle n = 5, Soya-I 0.5 μM n = 5; p = 0.0073 by unpaired *t* test; Figure 6C, G). In this hippocampal NPC cultures, GFAP-positive astrocytes were hardly detected (data not shown). 

Treatment with 1 μM soya-Ι in hippocampal NPCs for 4 days increased neurite length 2.2-fold more than the vehicle group (Vehicle n = 17, Soya-I 1 μM n = 17; p < 0.0001 by unpaired *t* test; Figure 6H, I). Primary branches growing directly out of MAP2-positive cells with neurites longer than double the cell body width and all secondary branches coming out of primary branches were counted. The numbers of both primary and secondary branches were also markedly increased in the soya-Ι-treated group (1 μM; Vehicle n = 17, Soya-I 1 μM n = 17; p<0.0001 by unpaired *t* test; Figure 6H, J).

**Figure 7:**
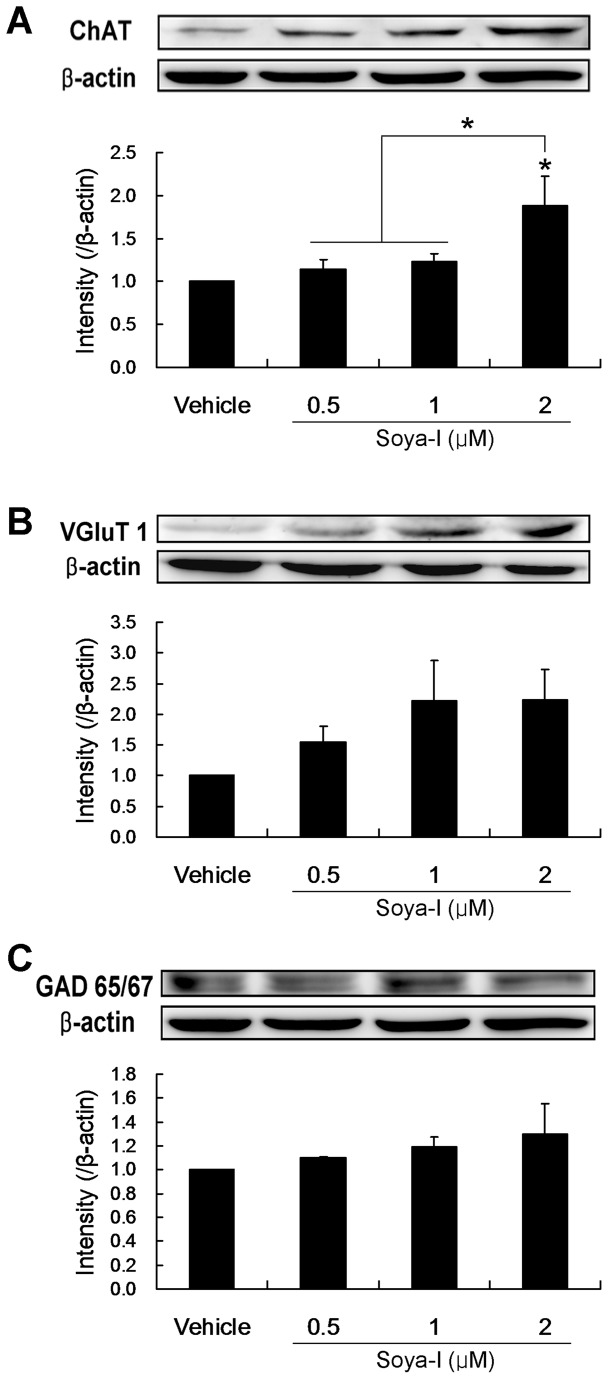
Effects of soya-Ι on expression of differentiation markers of neuronal cell types in cultured hippocampal cells. A. Effects of soya-Ι on the expression of ChAT protein. B. Effects of soya-Ι on the expression of VGluT1 protein. C. Effects of soya-Ι on expression of GAD 65/67 protein. All assay and measurements were carried out as described in Materials and Methods. Hippocampal cultures from the rat embryos were performed three times. Samples per groups were analyzed twice. Data represent means ± SEM (^*^ *p* < 0.05, compared with the vehicle group by Newman-Keuls Multiple Comparison Test).

When the cell extracts were analyzed by immunobloting after treatment with soya-Ι at 0.5, 1, and 2 μM for 6 days, the protein expression levels of ChAT (Figure 7A) were increased 1.9-fold, respectively, compared with those of the vehicle group (Vehicle n = 4, Soya-I 0.5 μM n = 4, Soya-I 1 μM n = 4; Soya-I 2 μM n = 4; F_3,12_ = 4.584, p = 0.0232 by One-way ANOVA). However, expression of VGluT1 or GAD65/67 proteins increased relatively little after treatment with soya-Ι for 6 days (Figure 7B, C). Thus, the results shown in Figures 6 and 7 demonstrate that soya-I increases the proliferation and differentiation of hippocampal NPCs in primary culture from embryos, supporting the idea that soya-I improves learning and memory in memory-deficient model rats most likely by promoting neurogenesis and maturation of hippocampal NPCs.

## Discussion

Neurogenesis facilitates the formation of hippocampal learning and memory, including episodic memory and spatial memory, which are impaired in degenerative brain diseases [1,2]. NPCs in the hippocampus of adult animals and humans are largely generated in the subgranular layer of the DG [2,37]. Adult DGCs, born by proliferation, subsequently undergo migration, fate determination, differentiation, and maturation to integrate into preexisting neural circuits [5,37,38]. However, most adult-born DGCs proportionally undergo apoptosis before integrating and developing in the subsequent maturation process [39]. Thus, the formation of memory may be associated with the proliferation and survival of adult-born DGCs [40].

In this study, we generated an animal model of hippocampal memory loss by damaging the entorhinal cortex. As reported previously, the entorhinal cortex is a major cortical input to the hippocampus and an important structure for memory formation [25,41]. In particular, pyramidal cells in layer III and stellate cells in layer II of the entorhinal cortex connect to the neurons of CA1 [41,42] and to the DG and CA3 subfields [43-45], and layer IV-VI cells of the entorhinal cortex and medial entorhinal cortex project to the DG of the hippocampus, respectively [46,47]. In this study, we induced neuronal damage by injecting IBO into three sites, including the entorhinal cortex and medial entorhinal cortex, as previously described [25,27]. As shown in Figure 3, neurons of the hippocampal CA1 and DG gradually degenerated because of their connections to the IBO-lesioned neurons in the entorhinal cortex by perforant pathways. In behavioral tests of cognitive functions, entorhinal cortex-lesioned animals displayed poor learning and memory abilities [25,48,49] and reduced expression of AChE [28], GAP-43, and synaptophysin [50] and showed decreased numbers of parvalbumin-expressing cells [49]. Many neuronal cells in the hippocampus and entorhinal cortex begin to die in the early stage of AD patient brain [25], resulting in memory loss and disorientation. We previously reported that neuronal cell types expressing VGluT1, GAD, and ChAT are significantly reduced in the hippocampus and entorhinal cortex of animals where IBO was injected into the entorhinal cortex [26]. 

Using the animal model of degenerating neuronal cells and memory loss, we observed that soya-Ι improves impairment in learning and memory in a series of behavioral tasks such as the Y-maze, passive avoidance, and Morris water maze tests. We also conducted several experiments designed to reveal the mechanism(s) underlying memory recovery. First, we showed that soya-Ι supports the proliferation and survival of adult-born DGCs in learning- and memory-impaired rats using the thymidine analog BrdU, which is incorporated into chromosomes with a short half-life (within 2 h *in vivo*), as a marker for cell proliferation [5,7,51,52]. This finding was further confirmed by the observation that soya-I directly acts on hippocampal NPCs cultured from the rat E16 embryonic brain by labeling with the Ki67 marker, which detects dividing cells except those in the G1 phase. Importantly, a previous report [53] found only 20 % of BrdU-positive cells 1 month after BrdU injection in the normal mouse hippocampus. However, we showed that approximately 30 % of BrdU-positive cells (48 ± 6.4 per hippocampal slice; Figure 2B) 1 week after soya-Ι administration (10 mg·kg^-1^) were maintained until 4 weeks later (13.8 ± 1.4; Figure 5B). Thus, we conclude that soya-Ι strongly promotes proliferation of hippocampal NPCs and supports the survival of newly born DGCs.

After proliferation, the adult-born DGCs continue differentiation, undergoing the phases of maturation and integration into the preexisting neuronal circuitry. Neurogenesis requires the coordination of intercellular inputs, such as glutamatergic and GABAergic inputs, throughout 2 - 4 weeks of early development after birth [2,5]. As shown in Figures 3 and 5, when we immunostained BrdU-positive, newly born DGCs with antibodies against VGluT1 and GAD67 at 4 weeks after BrdU injection, DGCs expressing GAD67 or VGluT1 increased to or even beyond that in the sham group with soya-I administration. In addition, the expression of VGluT1 protein was prominent in an immunoblotting assay of hippocampal NPCs at 6 days after treatment with soya-Ι. This suggests that some newly born DGCs have already differentiated to GABAergic or glutamatergic neurons, and excitatory glutamatergic input may play an important role in hippocampal learning and memory. In fact, Nakazawa and his collegues [54] reported that the excitatory glutamatergic input is associated with fast learning in one-time experiences and memory recall, mediated via NMDA receptors in Schaffer collateral - CA1 synapses. Thus, we suggest that neurogenesis caused by soya-I is associated with enhanced learning and memory in memory-deficient rats [1].

Synaptic connections in developing neurons contribute to dendritic arborization, including synaptic remodeling, during memory formation [55]. Indeed, in hippocampal NPCs cultured from the rat embryonic hippocampus, including precursor cells of pyramidal cells and granular cells, we observed progressive differentiation of NPCs, increased numbers of immature neurons, increased cells co-immunostained with NeuN, longer neurites, a higher number of dendrites, and more synaptic connections. Thus, we conclude that the effect of soya-Ι on synaptic remodeling may contribute to neuronal regeneration and memory formation. However, whether the expression of the NMDA receptor and activity-dependent synaptic plasticity mediated by the NMDA receptor are up-regulated by soya-I remains to be determined.

Memory loss and impairment are strongly correlated with reduced cholinergic function [3,4,56]. In particular, in patients with early AD, a leading cause of dementia [4], loss of hippocampus-dependent spatial memory is believed to be initiated by degeneration of cholinergic neurons [3,4]. In this study, we observed that the number of ChAT-positive cells in the adult rat hippocampus is increased more than 2-fold by oral administration of soya-Ι to learning- and memory-impaired rats and that the expression of ChAT protein is elevated by addition of soya-Ι to the cultures of hippocampal NPCs. However, we did not detect BrdU-positive DGCs that developed into cholinergic neurons (merged with ChAT) in the DG in our immunohistochemical assay at 4 weeks after soya-I administration. In the rodent hippocampus, ChAT-expressing cholinergic neurons are located primarily in the stratum lacunosum moleculare of the CA1 area, but there are few in the granular cell layer of the DG [57]. However, BrdU-positive cells born in the SGZ in the DG do not migrate to the stratum lacunosum moleculare region, but only migrate into the granular layer and become mostly GABAergic and glutamatergic cells. Thus, we may not be able to find any newly generated cells merged with ChAT in the DG, although ChAT-positive cells were still elevated approximately 2-fold at 4 weeks after soya-I administration. Further studies are required to investigate whether adult-born DGCs form synapses with and integrate into pre-existing cholinergic neurons. 

When Soya-I was administrated to IBO model rats, elevated neurogenesis was observed with 10 mg/kg soya beyond sham control levels (Figure 2), and the number of vGluT1-positive cells increased slightly more than that of the sham control group (Figure 5) although it was not determined as statistically significant when analyzed by one-way analysis of variance (ANOVA) followed with the Newman-Keuls multiple comparison test. This may be caused by neuronal damage induced by injection of IBO. Proliferation of neural stem cells and neurogenesis in DG and SVZ are stimulated to increase after experimental Traumatic Brain Injury (TBI) and ischemia [58,59]. This also suggests a possibility that soya-I may have effects on non-lesioned animals. However, memory abilities in any soya-I treated IBO model group were not elevated compared with the sham group in behavioral tests at 1 week and 4 weeks after administration (Figures 1 and 4). The effect of soya-I on normal animals remains to be investigated. In neural precursor cell cultures, the numbers of proliferating and differentiated cells were also higher in the soya-I treated group under the concentrations of 0.5 uM to 2 uM than vehicle groups. The upregulation of neurogenesis in cultures is difficult to compare directly to animal studies, but may indicate mechanisms by which Soya-I affects on maturing neurons and growing neurites. In addition, the comparability of dose between animal study and NPC cultures is not known. 

Finally, neurodegeneration is associated with inflammatory processes in the presence of injurious chemicals such as free radicals (e.g., superoxide and nitric oxide) and cytokines including interleukin-1 and tumor necrosis factor (TNF) [51,60]. Additionally, neuroinflammation and microglial pathology are crucial contributors to cognition and memory loss in many diseases related to memory dysfunction, such as AD [51,61]. In contrast, hippocampal neurogenesis and loss of memory function can be recovered by anti-inflammatory effects in the hippocampus [51]. An activated microglia cell marker, OX42-positive cells, decreased significantly in the DG and CA3 region of the hippocampus after soya-Ι administration. We previously reported that soya-Ι reduces the inflammatory response by inhibiting nuclear factor-κB activation in a colitic model mice. Soya-I also decreased inflammatory mediators, such as TNF-α, COX-2, iNOS, IL-1β in LPS stimulated mouse peritoneal macrophages [22]. Thus, soya-Ι probably facilitates anti-neuroinflammatory responses to neuroprotect from degeneration induced by IBO injection, in addition to neuroregeneration including neurogenesis and differentiation into cholinergic neurons in the hippocampal region of IBO-induced learning- and memory- impaired rats.

In conclusion, although memory-enhancing effects of soy have been reported previously [16-18,20,21,62], the active component(s) and mechanism(s) have not been clarified. Our results show that soya-Ι facilitated the recovery of learning and memory impairment by promoting neuronal regeneration processes including hippocampal neurogenesis, neurite outgrowth, number of dendrites of DGCs, and cell type differentiation in the hippocampal region, as well as supporting neuroprotection effects against neuroinflammation. 

## Supporting Information

Figure S1 **Loss of neuronal cell types in the entorhinal cortex of memory deficient model rats.** Neuronal cell types were immunostained using specific markers in ibotenic acid (IBO) induced model rats. (A) Cells expressing Glutamatergic neuronal marker, VGluT1 (green), (B) GABAergic neuronal marker, GAD67 (green) and (C) cholinergic neuronal marker, ChAT (green) were decreased in the rat entorhinal cortex by IBO injection. Nuclei were counter stained by propidium iodide (PI, red). (D) The percentage numbers of VGluT1, GAD67 or ChAT positive cells per microscopic filed in the saline (SAL) or IBO injected entorhinal cortex. Data represent means ± SEM (^**^ *p*<0.01, ^***^ *p* <0.001, compared with SAL group by unpaired *t* test).
(TIF)
