# Supplementary figures and images for: Soyasaponin I Improved Neuroprotection and Regeneration in Memory Deficient Model Rats
Source: PLoS One. 2013 Dec 4;8(12):e81556. doi: 10.1371/journal.pone.0081556 (PMC3852400; doi:10.1371/journal.pone.0081556)

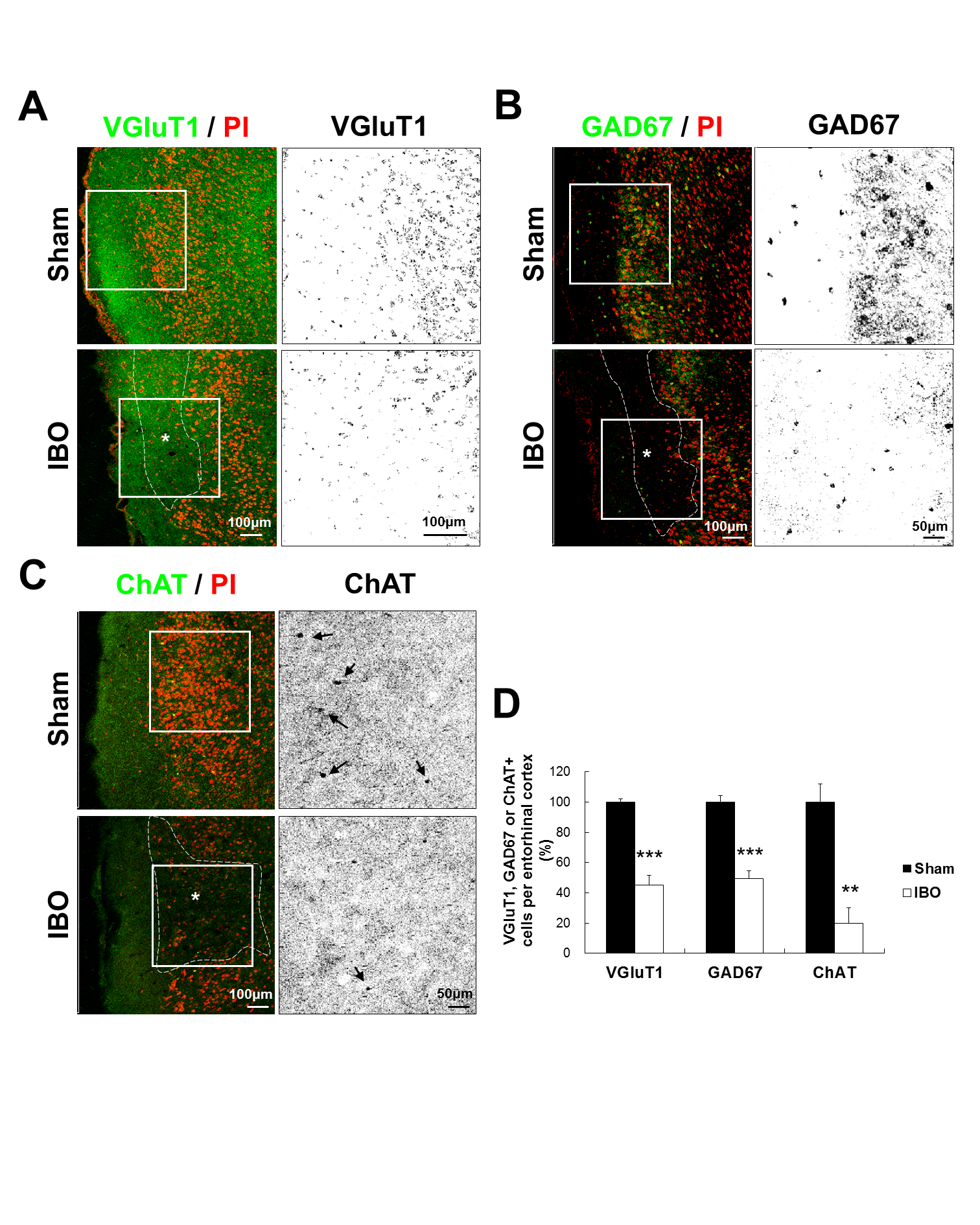

Supplement: Figure S1 — Loss of neuronal cell types in the entorhinal cortex of memory deficient model rats. Neuronal cell types were immunostained using specific markers in ibotenic acid (IBO) induced model rats. (A) Cells expressing Glutamatergic neuronal marker, VGluT1 (green), (B) GABAergic neuronal marker, GAD67 (green) and (C) cholinergic neuronal marker, ChAT (green) were decreased in the rat entorhinal cortex by IBO injection. Nuclei were counter stained by propidium iodide (PI, red). (D) The percentage numbers of VGluT1, GAD67 or ChAT positive cells per microscopic filed in the saline (SAL) or IBO injected entorhinal cortex. Data represent means ± SEM (** p<0.01, *** p <0.001, compared with SAL group by unpaired t test). (TIF) [file pone.0081556.s001.tif]
